# Purification and Characterization of Enterovirus 71 Viral Particles Produced from Vero Cells Grown in a Serum-Free Microcarrier Bioreactor System

**DOI:** 10.1371/journal.pone.0020005

**Published:** 2011-05-13

**Authors:** Chia-Chyi Liu, Meng-Shin Guo, Fion Hsiao-Yu Lin, Kuang-Nan Hsiao, Kate Hsuen-Wen Chang, Ai-Hsiang Chou, Yu-Chao Wang, Yu-Ching Chen, Chung-Shi Yang, Pele Choi-Sing Chong

**Affiliations:** 1 Vaccine Research and Development Center, National Health Research Institutes, Zhunan Town, Miaoli County, Taiwan; 2 Center for Nanomedicine Research, National Health Research Institutes, Zhunan Town, Miaoli County, Taiwan; 3 Graduate Institute of Immunology, China Medical University, Taichung, Taiwan; Indian Institute of Science, India

## Abstract

**Background:**

Enterovirus 71 (EV71) infections manifest most commonly as a childhood exanthema known as hand-foot-and-mouth disease (HFMD) and can cause neurological disease during acute infection.

**Principal Finding:**

In this study, we describe the production, purification and characterization of EV71 virus produced from Vero cells grown in a five-liter serum-free bioreactor system containing 5 g/L Cytodex 1 microcarrier. The viral titer was >10^6^ TCID_50_/mL by 6 days post infection when a MOI of 10^−5^ was used at the initial infection. Two EV71 virus fractions were separated and detected when the harvested EV71 virus concentrate was purified by sucrose gradient zonal ultracentrifugation. The EV71 viral particles detected in the 24–28% sucrose fractions had an icosahedral structure 30–31 nm in diameter and had low viral infectivity and RNA content. Three major viral proteins (VP0, VP1 and VP3) were observed by SDS-PAGE. The EV71 viral particles detected in the fractions containing 35–38% sucrose were 33–35 nm in size, had high viral infectivity and RNA content, and were composed of four viral proteins (VP1, VP2, VP3 and VP4), as shown by SDS-PAGE analyses. The two virus fractions were formalin-inactivated and induced high virus neutralizing antibody responses in mouse immunogenicity studies. Both mouse antisera recognized the immunodominant linear neutralization epitope of VP1 (residues 211–225).

**Conclusion:**

These results provide important information for cell-based EV71 vaccine development, particularly for the preparation of working standards for viral antigen quantification.

## Introduction

Enterovirus 71 (EV71) virus infection has recently caused outbreaks of hand-foot-and-mouth disease (HFMD) associated with severe neurological disease in young children [1], [2] and has become a serious public health problem in the Asia-Pacific region [1], [3]–[8] EV71 virus is a non-enveloped, single positive-stranded RNA virus of the family *Picornaviridae* with a genome size of approximately 7.4 kb. The EV71 virus is a pentameric icosahedral particle consisting of 60 copies of the VP1, VP2, VP3 and VP4 capsid proteins. Although the host receptors for EV71 virus had been identified [9], [10], there is no effective antiviral agent to combat EV71 infection. An effective vaccine is still the best strategy to control and prevent the disease. Experimental EV71 vaccines [8], [11] have included live-attenuated virus [12], [13], formaldehyde-inactivated virions [14], [15], virus-like particles [16], VP1 recombinant protein [17], [18], VP1 DNA vaccine [19], VP1 peptide-based vaccine [20], [21], bacterial or viral vectors expressing VP1 [17], [22], and a Vero cell-adapted live attenuated virus [13]. In previous murine immunogenicity studies, the formalin-inactivated EV71 virus produced from Vero cells elicited a more effective immune response than recombinant VP1 protein or DNA vector vaccines [17], [18]. These results are difficult to compare because the standard production methods, standard virus neutralization assays and methods used to quantify the critical viral antigens are not available. Therefore, the production of pure EV71 viral particles as a working standard is critical for EV71 vaccine development.

The semi-purified EV71 virion and EV71 virus like-particle (VLP) can be generated from the harvested virus concentrate by either precipitation with 30% polyethylene glycol or zonal ultracentrifugation using either a 40–65% discontinuous sucrose gradient or a 5–40% linear CsCl gradient [16], [18], [20], but information about the purity and physical structure of these virions is limited. In the present study, we describe the purification and biophysical characterization of EV71 viral particles that were produced from Vero cells grown in a serum-free microcarrier bioreactor system.

## Materials and Methods

### Ethics Statement

All experiments were conducted in accordance with the guidelines of Laboratory Animal Center of NHRI. The animal use protocols have been reviewed and approved by the NHRI Institutional Animal Care and Use Committee (Approved protocol No. NHRI-IACUC-098033-A).

### Cells, culture medium and virus

African green monkey kidney (Vero) cells were kindly provided by the Taiwan Centers of Disease Control (Taiwan CDC), which obtained the original Vero cell line (passage #125) from the American Type Culture Collection (ATCC). In general, Vero cells were grown in serum-free VP-SFM medium (GIBCO), and cells were passaged twice weekly in T-flasks. The E59 strain (genotype B4), the clinical isolate of the EV71 virus, was obtained from the Taiwan CDC. EV71/E59 virus stocks were collected from the supernatants of infected Vero cells at three days post infection (DPI). The titers of the virus stocks were determined by a plaque assay, and the stocks were stored at −80°C.

### EV71 virus cultivation using a bioreactor system

EV71 virus was cultivated using serum-free VP-SFM medium in a BIOFLO 310 bioreactor (NBS, US) based on the microcarrier cell culture bioprocess previously reported [15], [23]. Bioreactor culture medium containing 5 g per L Cytodex 1 was inoculated with 2×10^5^ cells per mL, and the cell density reached 2 to 2.5×10^6^ cells per mL after six days of cultivation. The Vero cells were infected with EV71/E59 at a MOI of 10^−5^. EV71/E59 virus stocks were collected from the microcarrier culture supernatants at 6 to 8 days post infection.

### EV71 virus purification by continuous sucrose gradient ultracentrifugation

The EV71 virus culture supernatant was harvested from the bioreactor culture. The cell debris was removed by passage through a 0.65 µm filter (Sartorius), and the supernatant was concentrated 20-fold with a 100 K TFF capsule (Pall). The crude EV71/E59 concentrate (∼200 mL from a 5 L run) was loaded onto a 10–50% continuous sucrose gradient and centrifuged at 32,000 rpm for three hours using a zonal rotor in a Hitachi CP80 ultracentrifuge. The fractions (50 mL per fraction) at 20 to 40% sucrose were collected and individually dialyzed against three exchanges of 1 L PBS at pH 7.4 (Gibco), then stored at 4°C. The infectivity of the purified virus fraction was assessed by a TCID_50_ assay. The fractions were also subjected to SDS-PAGE and western blot analyses. The zonal centrifugation-purified EV71/E59 virus was pooled and concentrated by diafiltration using an Amicon 100 K tube (Millipore) and centrifuged at 3,000× g, then stored at 4°C. The total protein concentration of the purified virus fractions was determined by a BCA protein assay. Half of the purified EV71 virus fractions (10 mL) was stored at −80°C in 0.5-mL aliquots; the other half was inactivated by 0.2% (v/v) formalin at 37°C for 3 days and stored at 4°C.

### Determination of viral titer

Viral titers were determined using the median endpoint of the tissue culture's infectious dose (TCID_50_). Serially-diluted virus samples (from 10^−1^ to 10^−8^) were added to Vero cells grown in 96-well plates, and 10 replicate samples were used for each dilution. The 96-well plates were incubated for six days at 37°C, and the TCID_50_ values were measured by counting the cytopathic effects (CPE) on infected Vero cells. The TCID_50_ values were calculated by the Reed-Muench method [24].

### SDS-PAGE analysis and Western blotting

SDS-PAGE analysis of the purified EV71/E59 virus fractions was performed in a NuPAGE 4–12% Bis-Tris Gel (Invitrogen) according to the protocol suggested by the manufacturer. For immunoblotting, EV71 viral proteins were directly electrotransferred onto the PVDF membrane. The membrane was subsequently soaked overnight at 4°C in 5.0% skim milk in PBS. Each membrane was incubated with PBS buffer containing one of two diluted (1∶1000) EV71-specific monoclonal antibodies: either MAB979 (Chemicon International) or E1 (produced in-house). Antibodies were bound for 2 hr at room temperature. The membranes were then washed five times with 15 mL assay buffer. Binding of the respective antibodies to the viral proteins was detected by adding 2 mL PBS buffer containing a horseradish peroxidase (HRP)-conjugated donkey anti-mouse secondary antibody (Jackson ImmunoResearch) at a dilution of 1∶10,000. After1-hr incubation at room temperature, the membrane was washed 6 times with assay buffer and blotted dry. The protein bands were revealed by adding TMB substrate solution (KPL).

### Mouse immunogenicity studies

Different concentrations of inactivated EV71 particles (5 and 25 µg) were adsorbed on 2 mL aluminum phosphate (3 mg of aluminum) at room temperature for 3 hr before immunization. One group of 6 female BALB/c mice (6–8 weeks old) were immunized intramuscularly (i.m.) with 0.2 mL of the alum-absorbed inactivated EV71 particles and were boosted twice with the same dose at two week intervals after priming. The immunized mice were bled one week after the final boost, and the serum was collected and used to analyze virus neutralization.

### Virus neutralizing assay

Serum samples were collected from immunized mice and inactivated at 56°C for 30 min. Each serum sample was added to a microtube and serially diluted two-fold with fresh cell culture medium; 400 µL of a 200-TCID_50_ virus suspension was then added to each tube containing 400 µL of serially diluted serum. After incubation at 4°C for 18–24 hr, 100 µL of serially diluted samples were added to the 96-well plates containing Vero cells. The cultures in the 96-well plates were incubated for 7 days at 37°C, and the TCID_50_ values were measured by counting cytopathic effects (CPE) in infected cells. The 50% neutralization inhibition dose (ID_50_) was calculated as the reciprocal of the serum dilution that yielded a 50% reduction in the viral titer using the Reed-Muench method.

### Characterization of EV71 viral particles by transmission electron microscopy (TEM)

Inactivated EV71 particles were deposited on a Formvar-coated and carbon-vaporized 200-mesh copper grid. The sample was kept on the copper grid for 15 min at room temperature, and excess sample was then removed with filter paper. After washing twice with ddH_2_O, the copper grid was stained with 2% phosphotungstic acid solution for 2 min, which was then removed with filter paper. The stained grid was dried for 3 days and observed under a Hitachi H-7650 electron microscope.

### EV71 viral RNA detection by real-time PCR

Viral RNA was isolated from 250 µL purified EV71 particles (40 µg total proteins by BCA analysis) using Trizol LS reagent (Invitrogen) according to the manufacturer's instructions. After precipitation, the RNA pellet was dissolved in RNA-Safeguard reagent solution (GeneMark, Taiwan). The first cDNA strand was synthesized in a reaction containing 6.6 µL dH_2_O, 0.5 mM dNTPs, 4 µL 5× buffer, 200 U SuperScript™ III reverse transcriptase (Invitrogen), 120 ng random primers (Invitrogen), 40 units RNaseOUT recombinant RNase inhibitor (Invitrogen) and 5 µL total RNA as the template material. The mix was incubated at 50°C for 1 h followed by 15 min at 70°C to denature the reverse transcriptase. Two units of *E. coli* RNase H (Fermentas, USA) were added and incubated at 37°C for 20 minutes to remove RNA complementary to the cDNA. The first cDNA strand product was then diluted 10-fold in water for the real-time PCR assay. A recombinant plasmid DNA carrying the EV71 VP1 gene was constructed for use in the standard curve for real-time PCR. The full-length VP1 sequence was amplified from the EV71-E59 genome by RT-PCR and cloned into the pGEM-T Easy Vector (Promega, USA). The recombinant plasmid was propagated in *Escherichia coli* DH5α competent cells and purified with the Plasmid DNA kit (BioKit, Taiwan); the concentration of the purified DNA was determined in a NanoDrop 2000c spectrophotometer (Thermo Fisher Scientific Inc., USA). The standard curve was generated from 10-fold serial dilutions of the recombinant plasmid from 2.2×10^8^ to 2.2×10^2^ copies. EV71 viral mRNA was assessed by quantitative PCR analysis with the LightCycler® 480 Real-Time PCR system and the EV71-specific primer pair EVVP1F: 5′-GGTACCCCACGTTTGGAGA-3′ (620–638 bp) and EVVP1R: 5′-TAGGACACGCTCCATACTCAAG-3′ (658–679 bp) and probe set No. 2: CAGGAGAA (Cat. No. 04684982001) from the Roche Universal Probe Library Assay Design Center (Roche). The real-time PCR was evaluated with the Maxima® Probe qPCR Master Mix (Fermentas, USA). Each reaction contained 1.0 µL 10-fold diluted cDNA, 0.1 µM each EVVP1F and EVVP1R, 0.05 µM probe, 10 µL master reaction mix, and water to a final volume of 20 µL. The data were analyzed with LightCycler Software.

### Peptide-ELISA

Fifty-seven overlapping synthetic peptides were synthesized using Fmoc chemistry by Kelowna International Scientific Inc. (Taipei Hsien, Taiwan) according to the sequence of the VP1 capsid protein of EV71. Each peptide contained 15 amino acids, 10 residues of which overlapped with the adjacent peptides. The reactivity of the antibody to each synthetic peptide was analyzed by an enzyme-linked immunosorbent assay (ELISA) according to the protocol previously reported by Panezutti *et al.* [25].

## Results

### EV71 virus production using a serum-free microcarrier bioreactor

To establish cell-based EV71 vaccine production, the previously reported serum-free microcarrier culture process was used to produce EV71 virus (18,34). Vero cells were amplified to 2.0–2.5×10^6^ cells/mL on 5 g/L Cytodex 1 microcarrier in either a 1.4-L or 5-L working volume bioreactor and infected by EV71 at a 10^−5^ multiplicity of infection (MOI). During the infection phase, the viral titers were determined by a TCID_50_ assay and found to reach 10^6^/mL at 6 days post infection (DPI); there was no increase in viral titers in the 7^th^ and 8^th^ DPI. The low MOI can result in slow kinetics of viral growth, but similar kinetics of viral growth based on the viral titer (TCID_50_) were found in both sizes of bioreactor. When the culture supernatants were harvested at either the 6^th^ or 8^th^ DPI, a 0.65-µm filter was very efficient for the removal of cell debris. In addition, 100 K and 300 K tangential-flow filter (TFF) capsules were tested for filtrate concentration; the 100 K TFF capsule was more effective at removing host cell proteins for downstream virus purification (data not shown).

### EV71 virus purification using sucrose gradient ultracentrifugation

**Figure 1:**
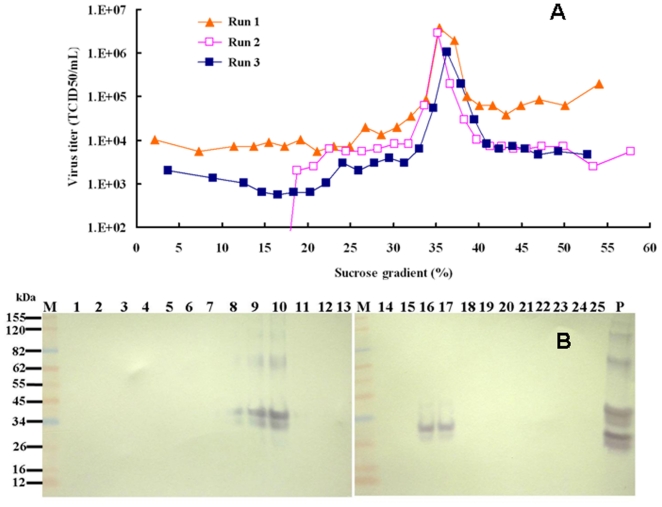
Purification of the EV71 virus by sucrose gradient zonal ultracentrifugation. The concentrated EV71 harvest stock was separated into 25 fractions. A. The viral titer of each fraction was determined by a TCID_50_ assay. B. The EV71 antigens were detected by western blot using MAb979. P means the positive control that is the formalin-inactivated EV71/E59 vaccine bulk produced by roller bottle method.

Zonal ultracentrifugation of a continuous sucrose gradient (10 to 50%) at 32,000 RPM for 3 hr was used to purify EV71 virus from the harvest concentrate described above. The viral titer and protein concentration of each fraction (50 mL) were determined by TCID_50_ and BCA assays, respectively. The results of three different experiments are summarized in Figure 1. The highest infectious viral titers were found in fractions 16 and 17 containing 35–38% sucrose, with viral titers reaching 5×10^6^ TCID_50_/mL (Fig. 1A). Viral titers in the range of 10^4^–10^5^ TCID_50_/mL were detected in other fractions. The viral proteins in each fraction were separated by SDS-PAGE, and the VP2-specific monoclonal antibody MAb 979 was used to verify the presence of EV71 virus by western blot. EV71 antigens were detected in two regions of the sucrose gradient (Fig. 1B). The first region that contained EV71 viral antigens was in fractions 8, 9 and 10, which contained 24–28% sucrose and had a low infectivity with a viral titer of approximately 10^3^ TCID_50_/mL. The second antigenic region co-located with the highest infectious EV71 viral fractions, 16 and 17, which had viral titers of 10^6^ TCID_50_/mL. The biochemical, viral and immunological properties of these two types of viruses (i.e., empty/defective viral particles or E-particles and infectious viral particles or F-particles) are defined below. To identify differences between these two types of EV71 viral particles, fractions 10 and 16 were individually diafiltrated with PBS and concentrated to 5 mL as virus stocks. The protein concentrations of the virus stocks of fractions 10 (E-particles) and 16 (F-particles) were 50 and 21.5 µg/mL, respectively. In six batches from the bioreactor, the protein concentration of the E-particles was consistently higher than that of the F-particles with a ratio of 7∶3.

### Biophysical characterization of EV71 viral particles by transmission electron microscopy (TEM)

**Figure 2:**
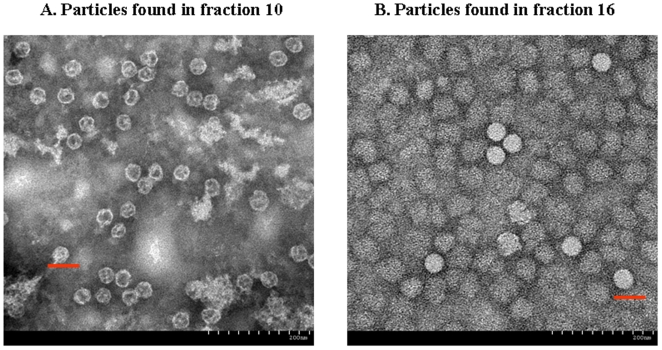
Photographs of EV71 viral particles as analyzed by transmission electron microscopy. (A) Fraction 10 was empty and had a defective particle (E-particle) structure. (B) Fraction 16 was full and had a solid particle (F-particle) structure. The bar represents 50 nm.

The physical structure of the EV71 viral particles in the virus stocks of fractions 10 and 16 were revealed by TEM analysis. For biosafety reasons, the purified virus stocks were individually inactivated by formaldehyde solution (4000∶1) at 37°C for 3 days. After preparation as described in the Materials and Methods, both samples were analyzed by TEM and found to have icosahedral particle structure (Fig. 2). The E-particles in fraction 10 (Fig. 2A) and F-particles (Fig. 2B) in fraction 16 were completely different. The diameters of the viral particles were approximately 30–31 nm and 33–35 nm for the E- and F-particles, respectively. The size of the F-particle is very similar to that of the enteroviruses of the *Piconaviridae* family [26]–[30]. In addition, TEM revealed that the physical structure of the E-particle was not solid, and the icosahedral capsid appeared to be empty (Fig. 2A). In contrast, the F-particles in fraction 16 were solid particles (Fig. 2B). Analyses described below demonstrated that these structural differences could be due to the presence of different viral proteins and RNA compositions.

### The viral protein composition of EV71 viral particles

**Figure 3:**
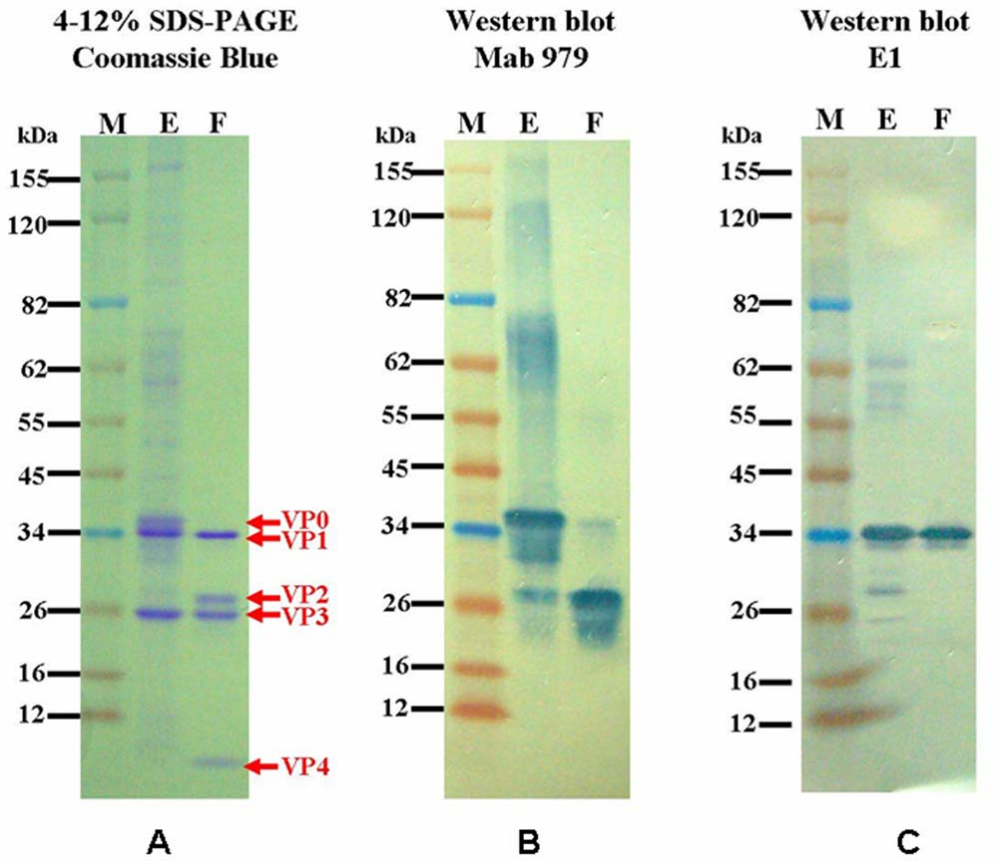
The viral antigen composition of the EV71 viral particle was analyzed by SDS-PAGE and western blot. (A) Two different types of EV71 viral particles were analyzed on a NuPAGE 4–12% Birs-Tris Gel. (B) EV71 viral proteins were detected by the MAb979 antibody. (C) EV71 viral proteins were detected by the E1 antibody.

Both the sucrose gradient zonal ultracentrifugation and TEM analysis demonstrated that there were two types of EV71 viral particles produced by Vero cells grown in the serum-free microcarrier bioreactor system. The viral proteins of the two EV71 viral particles were separated and analyzed by SDS-PAGE. The F-particles in fraction 16 contained four major protein bands with molecular weights (MWs) of 33 kDa, 28 kDa, 27 kDa and 8 kDa (Fig. 3A), corresponding to enterovirus capsid component proteins VP1 (33 kDa), VP2 (28 kDa), VP3 (27 kDa) and VP4 (8 kDa) based on their predicted protein sequences (Table 1). In-gel tryptic digest of the protein bands (33 kDa, 28 kDa, 27 kDa and 8 kDa) and analysis by MALDI-TOF spectrometry identified the tryptic fragments as human enterovirus 71 viral proteins based on sequences in NCBI Entrez.

**Table 1:** Summary of the predicted molecular weights (MW) of the EV71-E59 viral proteins and incomplete processed viral polypeptides [35].

EV71 antigen	Amino acid sequence	Residues	Predicted MW (kDa)
Viral antigen expected in the EV71 virion			
VP4	1–69	69	8
VP2	70–323	254	28
VP3	324–565	242	27
VP1	566–862	297	33
Incomplete processed viral polypeptides			
VP0 (VP4+VP2)	1–323	323	36
VP4+VP2+VP3	1–565	565	63
VP2+VP3	70–565	496	54
VP3+VP1	324–862	538	59
P1 (VP4+VP2+VP3+VP1)	1–862	862	95

The E-particles in fraction 10 contained three major protein bands with MWs of 36 kDa, 33 kDa and 27 kDa (Fig. 3A). The 36 kDa band was identified as incompletely processed VP2−VP4 (VP0) by mass spectrometry. Other high MW protein bands were also observed in fraction 10. To confirm the identity of the 36 kDa and high MW bands of the E-particle fraction, two EV71-specific monoclonal antibodies were used to identify the viral proteins by western blot. MAb979 is a commercially available monoclonal antibody with specificity against VP2 that recognized the 36 kDa band (VP0) and other high MW proteins in the E-particle fraction and a 28 kDa band in the F-particle fraction (Fig. 3B). These results suggest that the E-particles are composed of incompletely processed viral capsid proteins. A trace amount of the VP0 polypeptide was also found in the F-particle fraction by western blot (Fig. 3B). The E1 antibody is a VP1-specific monoclonal antibody produced in-house that reacted with the 33 kDa protein band in both fractions and some of the high MW protein bands (>55 to 62 kDa) in the E-particle fraction in the western blot analysis (Fig. 3C). Taken together, the results indicate that the two viral particles have different protein compositions. The western blot analyses indicated that the E-particle fraction was composed of the incompletely processed VP1-related and VP2-related proteins. In addition, the high MW proteins in fraction 10 that did not react with MAb979 and E1 may be contaminants derived from host cell proteins and are now being characterized in our laboratory.

### RT-PCR determination of the EV71 viral RNA content

**Figure 4:**
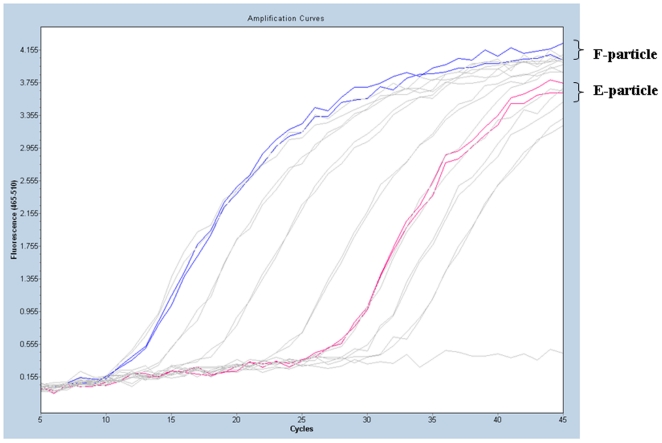
EV71 viral RNA content measured by RT-PCR. The results of quantitative RT-PCR using primers specific for a 60 bp region of the EV71 VP1 gene are reported for the F-particle and E-particle by the blue line and red line, respectively.

As described above, the E-particle had low virus infectivity, contained incompletely processed viral polypeptides, and was considerably lighter than the F-particle based on the sucrose gradient ultracentrifugation. Viral RNA was individually extracted from both EV71 viral particles to confirm that the differences in physical structure (empty and solid) of the two types of EV71 particles were due to the RNA contents and packaging. The EV71 RNA contents were measured by quantitative RT-PCR using specific primers to amplify a 60 bp region of the VP1 gene (Fig. 4). The signal for the VP1 RNA of the F-particle (blue line in Fig. 4) was detectable after 12 cycles of PCR. In contrast, the E-particle (red line) gave a detectable signal only after 28 PCR cycles. The approximate difference in the cycling threshold (Δ*C*_t_) value was at least 16, and therefore there was at least a 65536-fold difference between the RNA contents of the F-particle and E-particle, indicating that the E-particle had a lower EV71 viral RNA content than the F-particle. The low RNA content of the E-particle is consistent with its low infectivity and empty physical appearance in the TEM analysis.

### Differences in virus packaging

**Figure 5:**
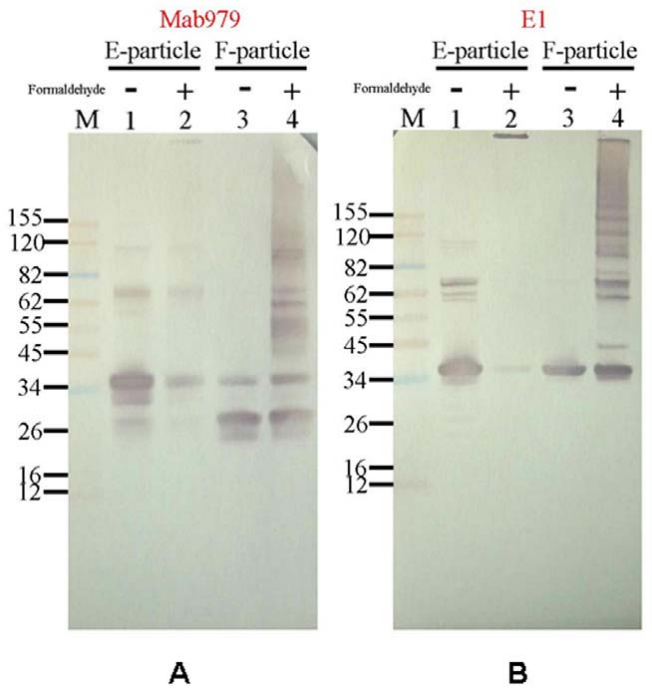
Western blot analyses of EV71 viral particles inactivated by formaldehyde. The two different types of formalin-inactivated EV71 particles were separated on a NuPAGE 4–12% Bis-Tris Gel and analyzed by two monoclonal antibodies: (A) the MAb979 antibody and (B) the E1 antibody.

The two EV71 viral particles were inactivated with formaldehyde (formalin) solution (4000∶1), and modifications were detected by western blot analysis. After formalin inactivation, the pattern of EV71 viral proteins in each viral particle was revealed using MAb979 and E1 monoclonal antibodies (Fig. 5). In the formalin-treated E-particle, both antibodies revealed that the viral proteins were cross-linked to form high MW protein complexes. In particular, the Western blot derived from E1 antibody showed that VP1-related proteins had extensive cross-linking (Fig. 5B, lane 2), and most likely the pentamer was covalently cross-linked together because no protein bands below 200 kDa MW were observed. In the formalin-inactivated F-particle, both MAB979 and E1 antibodies bound to VP1 and VP2 viral proteins within the pentamer that were cross-linked to form diverse high MW protein complexes (Fig. 5A&B, lane 4). The differences in the cross-linking patterns revealed by monoclonal antibodies also suggest that there are different conformations within the pentamer of the immature defective E-particles.

### Mouse immunogenicity studies of EV71 viral particles

We next investigated whether these two types of formalin-inactivated viral particles generated different types of immune responses. Five groups of mice were immunized with different amounts of the inactivated particles formulated with 300 µg Alum and boosted with the same dosage after 2 weeks. With the exception of group A (PBS control), antisera from all of the groups of mice had virus neutralization titers against the EV71/E59 virus (Table 2). The formalin-inactivated F-particle induced a higher virus neutralizing antibody response in the mouse model than did the inactivated E-particle. The current results from groups D & E (Table 1) indicate that 1 µg of F-particle may induce the maximum virus neutralizing antibody responses in the mouse model. The low dosage (0.5 μg) of the E-particle (group B) elicited a 5- to 10-fold lower virus neutralization titer than was observed in the other groups (p<0.05). At the high dose (2.5 μg), the defective EV71 viral particle induced a virus neutralizing antibody response that was almost 2-fold less potent than that induced by the low dosage of the F-particle (groups C and D in Table 2). Therefore, we conclude that the formalin-inactivated F-particle is more potent than the E-particle in the virus neutralizing antibody responses.

**Table 2:** EV71 virus neutralization titers of mouse antisera generated against two different types of EV71-E59 viral particle fractions as measured by a TCID_50_ neutralization assay.

Group	Sample	Total protein (µg)	TCID_50_ neutralization titer (GMT ± SE)
A	PBS	0	<20
B	E-particles	0.5	188±105
C	E-particles	2.5	906±351
D	F-particles	0.5	1604±533
E	F-particles	2.5	2020±799

### Linear immunodominant epitope mapping

We also evaluated whether the two types of particles generated a different spectrum of antibodies that recognized different linear immunodominant epitopes. The antisera of groups C and E were screened by peptide-ELISA for their reactivity with 57 overlapping synthetic peptides that covered the entire VP1 protein. Both antisera reacted solely with the VP1-43 peptide corresponding to residues 211–225 (FGEHKQEKDLEYGAC), which was previously identified as the immunodominant linear neutralization epitope in other studies [20], [21]. Although the immune potency of the two viral particles is different, they generate antibodies that recognize the same linear immunodominant neutralization epitope with similar titer (>1/12,800) in the mouse model.

## Discussion

Several experimental EV71 vaccine candidates are being developed, and encouraging mouse immunogenicity results have been obtained when the prototype vaccine candidates were tested at the preclinical level [for reviews see references [8] and [11]]. Currently, inactivated whole-virion EV71 vaccines appear to be the most potent vaccine candidates; however, immunological results are difficult to compare because the standard production methods, standard virus neutralization assays and the methods used to quantify the critical viral antigens are not available. The whole virion EV71 vaccine was produced by very crude methods, and the downstream purification process and the risk of contamination by host cell proteins were not investigated [14], [15], [23]. Therefore, the biochemical, biophysical and immunological characterizations of purified EV71 viral particles were performed in this study. In addition, we also evaluate whether the purified EV71 viral particles could be used as working standards for EV71 vaccine development. In the current study, a serum-free microcarrier Vero cell culture system for EV71 virus production was established using a low multiplicity of infection (MOI) to minimize virus seed usage, which could easily be adapted to a large-scale bioreactor process. The low MOI (10^−5^) did not reduce the EV71 viral titer (5×10^6^ TCID_50_). Serum-free medium was used to avoid problems associated with bovine serum, such as lot-to-lot inconsistency, potential prion contamination and the difficulty of removing the high serum protein content during downstream purification steps.

In this study, two types of EV71 viral particles (E- and F-particles) were produced from a serum-free Vero cell microcarrier bioreactor system and separated and purified using continuous sucrose gradient ultracentrifugation. These results are very similar to those of poliovirus studies in which two different polio viral particles were isolated and identified by sucrose density centrifugation [29]. Two types of poliovirus structures (D- and C-antigens) were observed and characterized by electron microscopy and biochemical assays. The D-antigen, like the F-particle in this study, had a high viral RNA content and a full particle structure. In contrast, the C-antigen lacked RNA content and had an empty particle structure [28] like the E-particle found in the current study. When we compared the total protein yield of these EV71 viral particles from the six batches from the bioreactor, the ratio of the E-particle to the F-particle was consistently 7∶3. We are currently investigating the parameters (altering MOI, culture conditions, harvest times, etc.) of the Vero cell culture that influence the formation of the E-particle.

The two EV71 particles had similar icosahedral structures, but their sizes were slightly different, 31–33 nm and 33–35 nm for the E-particle and F-particle, respectively. This size difference is most likely due to differences in the composition of the viral protein components and the viral RNA contents as described above. Generally, the morphogenesis of the *Picornaviridae* virus begins with the freshly translated P1 polypeptide forming the promoter for self-assembly into a pentamer unit, followed by formation of the empty capsid shell by additional pentamers. The pre-virion and virion formation requires the specific cleavage of the P1 polypeptide [31]. The P1 polypeptide is cleaved into the VP0, VP1 and VP3 proteins by the viral non-structural protein, 3CD protease. The VP0 protein is then cleaved into VP2 and VP4 by autocatalytic action that involves the viral RNA [26]. Based on the SDS-PAGE and western blot analyses, the E-particle was an immature particle in which the VP0 protein (VP2+VP4) was incompletely processed. The components of the E-particle were very similar to those of the C-antigen of poliovirus [31]. Some high MW protein bands (60 to 70 kDa) were detected in the E-particle, indicating that the P1 polypeptide was most likely incompletely processed during viral assembling and packaging. In addition, potential cleavage sites were predicted in the P1 polypeptide that could generate different sizes of viral proteins [32]; http://www.cbs.dtu.dk/services/NetPicoRNA/). Our results indicate that the immature capsid constructed by incompletely processed viral proteins can still form the icosahedral particle structure. The trace amount of VP0 found in the F-particle fraction supports cleavage of the VP0 protein into VP2 and VP4 by autocatalytic action involving viral RNA [26]. Quantitative RT-PCR studies of the VP1 gene content suggest that the EV71 viral RNA was packaged into the immature capsid to form the defective E-particles. The approximate difference in the cycling threshold (Δ*C*_t_) value was 16, indicating there was at least a 10,000-fold lower RNA content in the E-particle than in the F-particle. The low RNA content of the E-particle is consistent with its low infectivity and the empty physical appearance observed by TEM.

Formalin inactivation was performed to further confirm the differences in the physical structures of the two types of EV71 particles for vaccine production. Formalin-inactivated vaccines have been effective by cross-linking the primary amino groups in the proteins with aldehyde and other nearby nitrogen atoms. The different cross-linking patterns of the capsid proteins after formalin inactivation suggest that the conformations and interactions of the viral proteins within the pentamer of the E- and F-particles are likely different. This difference in particle structure and the interactions between the viral proteins are not easily observed by TEM. Differences in conformation were also observed in non-infectious empty particles and infectious viral particles of type 3 poliovirus [27].

The ability of the formalin-inactivated defective EV71 E-particle to induce neutralizing antibody responses in the mouse immunogenicity study is very different from that of the poliovirus C-antigen. The C-antigen could not induce neutralizing antibodies in poliovirus vaccine murine immunogenicity studies [27], [30]. The particle configuration of the C-antigen was different than that of the D-antigen, which influenced the display of antigenic sites [27]. The main antigenic sites have been identified as conformational and are located in the VP1, VP2 and VP3 regions of poliovirus [33]. These findings are also different from EV71 virus. The important murine immunodominant linear epitopes of EV71 have been identified as residues 208–222 and 240–260 of the VP1 capsid protein [20], [21], [34]. The synthetic peptides SP55 and SP70 corresponding to residues 163–177 and 208–222 of VP1, respectively, elicited neutralizing antibodies against EV71 infection [20], [21]. The neutralizing antibodies induced by the formalin-inactivated E-particles were less potent than the formalin-inactivated F-particle in a virus neutralization assay, but interestingly antibodies generated from both particles recognized the same immunodominant linear neutralization epitope of VP1 reported previously (residues 211–225). The results indicate that the configuration of the EV71 E-particle may not be different enough to influence the important antigenic and immunogenic sites. To be cost-effective the E-particles of EV71 could be considered as an additional antigen to the F-particles for EV71 vaccine development since the content of E-particles is at least 2-fold more than the F-particles in the final production yield. In conclusion, the current findings and the full characterization of the EV71 viral particles provide valuable information for the development of cell-based formalin-inactivated EV71 vaccine, in particular the F-particles could be used in a standardized assay for EV71 antigen quantification.
